# The Interaction of Central Nervous System and Acute Kidney Injury: Pathophysiology and Clinical Perspectives

**DOI:** 10.3389/fphys.2022.826686

**Published:** 2022-03-04

**Authors:** Yiru Wang, Siyang Liu, Qingquan Liu, Yongman Lv

**Affiliations:** ^1^Department of Nephrology, Tongji Hospital, Tongji Medical College, Huazhong University of Science and Technology, Wuhan, China; ^2^Health Management Center, Tongji Hospital, Tongji Medical College, Huazhong University of Science and Technology, Wuhan, China

**Keywords:** central nervous system, acute kidney injury, paraventricular hypothalamic, sympathetic nervous system, neuromodulation

## Abstract

Acute kidney injury (AKI) is a common disorder in critically ill hospitalized patients. Its main pathological feature is the activation of the sympathetic nervous system and the renin-angiotensin system (RAS). This disease shows a high fatality rate. The reason is that only renal replacement therapy and supportive care can reduce the impact of the disease, but those measures cannot significantly improve the mortality. This review focused on a generalization of the interaction between acute kidney injury and the central nervous system (CNS). It was found that the CNS further contributes to kidney injury by regulating sympathetic outflow and oxidative stress in response to activation of the RAS and increased pro-inflammatory factors. Experimental studies suggested that inhibiting sympathetic activity and RAS activation in the CNS and blocking oxidative stress could effectively reduce the damage caused by AKI. Therefore, it is of significant interest to specify the mechanism on how the CNS affects AKI, as we could use such mechanism as a target for clinical interventions to further reduce the mortality and improve the complications of AKI.

**Systematic Review Registration:** [www.ClinicalTrials.gov], identifier [registration number].

## Introduction

Acute kidney injury (AKI) is a complex syndrome marked by sudden decrease in glomerular filtration rate (GFR). Generally, the disease shows a slight and sustained elevation in serum creatinine concentration, oliguria or anuric renal failure (Levey and James, 2017). According to the anatomical location of primary cause, the etiologies of AKI are roughly classified into three categories: pre-renal, intra-renal, and post-renal origin (Kellum et al., 2013). The hall marks of AKI pathophysiology include renal microvasculature injury (Maringer and Sims-Lucas, 2016), maladaptive repair (Ferenbach and Bonventre, 2015) and activation of neurohumoral mechanisms, like sympathetic nervous system (SNS), renin–angiotensin system (RAS), systemic inflammatory response and oxidative stress (Grisk, 2020; Grivei et al., 2020; Legrand and Bokoch, 2021). Under normal conditions, interaction between nervous system and kidney is reasonable to maintain a normal physiological balance. Activation of central nervous system (CNS) and RAS promotes the maintenance of renal homeostasis osmolality, sodium balance and renal blood flow by regulating the secretion of vasopressin and modulating efferent renal sympathetic nerve activity (ERSNA) to induce renin secretion and decrease urinary sodium excretion (Tanaka and Okusa, 2020). Under pathological conditions, such as hypertension, AKI and chronic kidney disease (CKD), the interaction between the nervous system and the kidney could be out of balance. Such imbalance further leads to loss of normal homeostasis. Damaged kidneys usually involve acute activation of the SNS, which in turn may further accelerate the loss of renal function. These adaptive changes eventually become a deleterious factor for the progression of kidney damage (Zhang et al., 2018; Cao et al., 2020).

At present, clinical treatment of AKI was primarily symptomatic and supportive treatment, including dialysis, renal replacement therapy (RRT), and fluid administration according to personal circumstances. There was no specific therapy for rapid recovery from AKI. That was the reason why the mortality of AKI remained high. Current AKI therapy primarily focused on dysfunction, cell death, oxidative stress and inflammation of renal tubule epitheliums (Aparicio-Trejo et al., 2020). In recent years, researchers on AKI therapy targets devoted their efforts in fields concerning the neuroimmune system (Inoue et al., 2016). The activation of the CNS caused by AKI has been shown to relate to more severe renal impairment (Campese and Kogosov, 1995). Furthermore, AKI has deleterious effects on the CNS (e.g., uremic encephalopathy and stroke) and this disease can result in higher mortality, lower quality of life and higher health care costs (Nongnuch et al., 2014). Therefore, modulating the activation of CNS in AKI might be a new and promising therapeutic target. The purposes of this review were to describe the mechanisms of altered CNS pathways due to AKI and to consider the potential therapeutic implications.

## Kidney and Neuromodulation

### Renal Related Neuromodulation Under Normal Conditions

Kidney is densely under control by afferent renal sensory nerve and efferent renal sympathetic nerve that run in the outer layer of renal artery and around kidney (Tellez et al., 2013). There is interaction between the renal afferent and efferent nerves. The pathway is mediated by norepinephrine (NE), which increases and decreases afferent renal nerve activity (ARNA) by activating renal α_1_-and α_2_-adrenoceptors, respectively (Kopp et al., 2011). The renorenal reflex is a negative feedback loop to maintain renal function in a physiological state, consisting of a combination of renal afferent and efferent nerves (Larsen et al., 2014). ERSNA increases renin secretion rate and renal tubular sodium reabsorption, and it reduces renal blood flow while increasing ARNA (Colindres et al., 1980). Activation of ARNA triggers inhibitory renorenal reflex and decreases ERSNA. Those activities would lead to diuresis and minimize sodium retention (Kopp et al., 2007; Ma et al., 2008; Johns et al., 2011).

Previous study showed that ARNA has a predominantly inhibitory effect in normal rats (Colindres et al., 1980). Renal afferent sensory nerves are activated by renal pelvic pressure as well as stimuli such as NE, producing an inhibition on ERSNA to minimize sodium retention (Kopp et al., 2011). This response to renal afferent sensory nerves is modulated by sodium intake. The mechanisms include increased activation of angiotensin II type 1 (AT_1_) receptors and α2 adrenergic receptors under low sodium diet conditions, thereby inhibiting ARNA, and increased activation of endothelin-B receptors under high sodium diet conditions, thereby enhancing the responsiveness of afferent renal sensory nerves (Kopp, 2015). In summary, in healthy individuals, afferent sensory nerve, efferent sympathetic nerve and the kidney work together to keep body sodium and water balance by inhibitory renorenal reflex pathway in response to varying sodium intake.

### Renal Related Neuromodulation in Acute Kidney Injury

Acute kidney injury is defined as an abrupt decrease in renal function resulting from a combination of multiple factors (Makris and Spanou, 2016). Renal ischemia-reperfusion injury (IRI) is one of the most common causes of AKI in hospitalized patients. Animal studies have shown that the inhibitory renorenal reflex pathway is impaired in the IRI model, and that the absence of the inhibitory renorenal reflex leads to an inappropriate increase in ERSNA (Kopp and Buckley-Bleiler, 1989). Increased ERSNA stimulates the release of renin from the juxtaglomerular cell, resulting in increased production of angiotensin II (Ang II), which in turn triggers renal vasoconstriction (Naito et al., 2008). Renal vasoconstriction leads to a decrease in renal blood flow and GFR, thereby aggravating ischemic renal damage (Stefanska et al., 2016). In addition, Ang II also stimulates aldosterone release, which strongly enhances sodium and water reabsorption in the distal tubule and the collecting duct, leading to sodium and water retention (Satou et al., 2020).

Increased ERSNA leads to increased release of plasma NE (Fujii et al., 2003). In recent years, NE was found to be associated with inflammation and progressive tissue injury after AKI. Increased ERSNA during ischemia and post-reperfusion renal venous NE overflow plays a significant role in the development of ischemic AKI. A study of rat IRI model showed that plasma NE levels were significantly elevated after AKI and the levels were differed by sex. More specifically, male rats had a more pronounced increase than females did the degree of renal histological damage was also significantly different, which correlated with NE levels (Tanaka et al., 2013). Tyrosine hydroxylase (TH) is the rate-limiting enzyme for NE synthesis. Other studies also found increased expression of TH in IRI model (Cronin et al., 1978; Bellomo and Giantomasso, 2001). In addition, a study showed that renal NE levels remained elevated until day 16 after unilateral renal ischemia-reperfusion, whereas renal denervation significantly reduced renal NE expression and attenuated apoptosis and tubular injury. The study also revealed that denervated kidneys had a significant reduction in GFR after NE treatment as compared with intact kidneys (Kim and Padanilam, 2015). Early experimental studies showed that renal arterial infusion of norepinephrine could induce ischemic AKI in rats by causing renal vasoconstriction (Conger et al., 1991). In conclusion, evidence from experimental studies suggested that renal sympathetic activation might play a major role in the development and progression of AKI.

## Modification of Central Pathways

### Renal Afferent Sensory Nerves Regulate Sympathetic Activity in the Central Nervous System

As an important sensory organ, the kidney has abundant baroreceptors and chemoreceptors, as well as a large number of afferent sensory innervation. It communicates with the CNS via afferent sensory nerves (Webb and Brody, 1987). Renal baroreceptors sense alterations in renal perfusion and intrarenal pressure, and renal chemoreceptors receive stimuli from ischemic metabolites or uremic toxins (Koepke and DiBona, 1985). Renal afferent nerves have been shown to project directly to regions of the CNS such as the lateral hypothalamic area, the paraventricular nucleus (PVN), and indirectly to other areas of the hypothalamus (Calaresu and Ciriello, 1981). When the renal afferent nerve receives adverse stimulation due to the increase of adenosine and other metabolites caused by ischemia, ERSNA will reflexively increase (Katholi et al., 1984). Related studies have shown that renal injury activates renal afferent sensory nerve signals, which activate norepinephrine neurons in the posterior hypothalamus, lateral hypothalamus and the locus coeruleus (LC) (Campese and Kogosov, 1995). Notably, among brainstem noradrenergic neurons, the LC is the largest source of norepinephrine production (Chandler, 2016). In contrast, Selective renal afferent block inhibit the increase in NE levels in the posterior hypothalamus and the LC by cutting the dorsal root of T10-L2 without damaging the efferent sympathetic nerve (Campese and Kogosov, 1995). The findings further demonstrated that renal afferent nerve would activate the CNS and enhance sympathetic activity.

The hypothalamus plays a crucial role in the regulation of fluid homeostasis, with the PVN being a crucial brain region in the hypothalamus (Badoer, 2010). The PVN is located near the third ventricle and is an essential integrated site involved in endocrine and neural control. PVN consists of two types of neurons, magnocellular neurons and parvocellular neurons. The magnocellular neurons project to the posterior pituitary gland and release arginine vasopressin (AVP), which are involved in the regulation of fluid homeostasis (Swanson and Sawchenko, 1983), while the parvocellular neurons play a prominent role in the regulation of sympathetic nerve activity (Shafton et al., 1998).

### Mechanisms of Renal Afferent Sensory Nerve Modulation in the Central Nervous System

It has been confirmed that during stimulation of the afferent renal nerve, the firing frequency of magnocellular neurons in the PVN increases (Ciriello, 1998). Stimulation of magnocellular neurons in the PVN by the afferent renal nerve projects to the pituitary gland, resulting in AVP release upon afferent renal nerve activation. Electrical stimulation of the afferent renal nerve increases the firing rate of PVN neurons that project directly to the rostral ventrolateral medulla (RVLM). It suggested that stimulation of afferent renal sensory nerves might lead to activation of neurons within the PVN (Nishi et al., 2017). The RVLM is a decisive nucleus in sympathetic regulation. The majority of renal sympathetic premotor neurons are located in the RVLM the most represented neurons are catecholamine synthesizing cells in the cell population of the RVLM (Ding et al., 1993). Studies have confirmed that electrical stimulation of renal afferent nerve causes the activation of TH-positive containing neurons in the RVLM and non-TH neurons in the NTS (Nishi et al., 2017).

Different neurons in the PVN play different roles, with pre-sympathetic neurons mediating sympathetic excitation and GABAergic or NO-producing interneurons mediating sympathetic inhibition (Affleck et al., 2012). Treatment of ischemic AKI rats with intravenous GABA inhibited the enhancement of ERSNA during ischemia and the increase of NE overflow after reperfusion (Kobuchi et al., 2009). There are two known GABA receptor subtypes: GABA_*A*_ and GABA_*B*_. The GABA_*A*_ receptors work by coupling to the ligand-gated Cl^–^channel, and the GABA_*B*_ receptors are the archetype of heterodimeric G protein-coupled receptors (Johnston, 1996). Further studies demonstrated that the protective effect of intravenous GABA on AKI is mediated by inhibition of central sympathetic outflow, and the prevention of the AKI long-term kidney dysfunction is achieved through activation of GABA_*B*_ rather than GABA_*A*_ receptors (Kobuchi et al., 2015). Furthermore, removal of the common renal nerve, which includes afferent sensory nerves and efferent sympathetic nerves, increased GABAergic neuronal activity in the PVN (Chen et al., 2016). This suggested that the improvement in GABAergic neurotransmission in the PVN is partly due to the absence of afferent renal sensory nerve.

**FIGURE 1:**
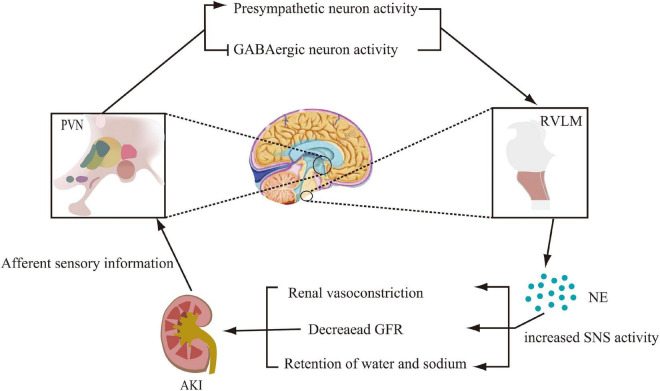
Modification of the central nervous system pathways involved in sympathetic activation during acute kidney injury (AKI). Activation of renal afferent sensory nerves, which project to the paraventricular nucleus (PVN) of the hypothalamus, promotes pre-sympathetic neuronal activity and inhibits GABAergic neuronal activity. By projecting from the PVN to norepinephrinergic neurons in the rostral ventrolateral medulla (RVLM), norepinephrine (NE) levels are increased, thereby promoting sympathetic nervous system (SNS) activity, which further leads to renal vasoconstriction, decreased glomerular filtration rate (GFR), and sodium and water retention.

The above experimental studies allowed us to reasonably speculate that AKI stimulates PVN pre-sympathetic neuronal activity through activation of afferent renal nerves in processes including ischemia and metabolites. The stimulus then activates catecholaminergic neurons in the RVLM and enhances renal sympathetic activity. These would cause renal vasoconstriction, decrease GFR, and exacerbate renal tissue injury (Figure 1). However, additional exploration of the underlying mechanisms was still needed.

## The Role of Renin–Angiotensin System

Since activation of the renin-angiotensin system (RAS) is strongly up regulated in AKI, it is particularly important to understand the mechanisms involved. Previous studies have shown that activation of the intrarenal RAS is mediated by overexpression of the brain RAS, which in turn activates the SNS (Campese et al., 2002). Under physiological conditions, Ang II regulates the RAS through negative feedback and inhibits renin release directly (Johns et al., 1990). However, in acute kidney injury, Ang II plays an indirect role in promoting the release of renin through sympathetic activation, thus forming a positive feedback pathway (Hering and Winklewski, 2017). Ischemic AKI mice showed increased brain TH synthesis and increased renal NE levels, indicating increased central sympathetic drive and enhanced ERSNA (Cao et al., 2017). PVN and RVLM are the sites of response that regulates ERSNA in renal IRI, and Ang II in this region acts via the oxidative stress pathway (Seifi et al., 2014). Ang II stimulates the AT_1_ receptors of PVN and RVLM to activate intracellular pathways, thereby stimulating NAD(P)H oxidase to mediate reactive oxygen species (ROS) synthesis to increase sympathetic outflow (Gao et al., 2004). Ang II was found to bind to the AT 1 receptor in the PVN to activate c-Src, which then modulates the effects of Ang II on sympathetic activity by activating NAD(P)H oxidase and subsequent superoxide anion production in the PVN. Microinjection of Ang II into the PVN increased c-Src activity, NAD(P)H oxidase activity and superoxide anion levels in the PVN of normal rats (Li and Pan, 2005; Han et al., 2011). Central sympathetic excitation during ischemic AKI is clearly dependent on the central RAS and ROS. In the ischemic AKI model, inhibition of central RAS/ROS with central RAS inhibitors or antioxidants prevented intrarenal RAS activation, decreased renal NE and reduced IRI-induced renal injury by approximately 60% (Cao et al., 2017), which demonstrated the importance of the central RAS/ROS activation pathway in AKI. Interestingly, the study also found that capsaicin treatment interrupted renal afferent innervation or renal denervation, inhibited brain-renal RAS axis activation and improved renal injury, implying that afferent renal sensory nerves play an important role in central sympathetic nerve excitation and renal injury. In addition, Ang II also exerts a regulatory effect on GABAergic neurons in the PVN. Ang II activates presynaptic AT-1 receptors, which increase the firing of PVN-output neurons projecting to the spinal cord and RVLM by inhibiting GABA release (Li and Pan, 2005). ROS, particularly superoxide anion, is closely associated with presynaptic modulatory signaling by Ang II on GABAergic synaptic inputs to pre-sympathetic neurons of the PVN, mediated by G_*i/o*_ proteins (Chen and Pan, 2007). In conclusion, the brain-renal RAS axis is an important part of the mechanism of renal injury caused by AKI, but its complete mechanism remains unclear.

## Mechanism of Renal-Brain Inflammation in Acute Kidney Injury

Acute kidney injury is a systemic inflammatory response process. It involves activation of pro-inflammatory cytokines and chemokines and infiltration of neutrophils and macrophages (Bonventre and Zuk, 2004). Those processes could further exacerbate kidney injury and distant organ damage (Doi and Rabb, 2016). In a mouse model of renal ischemia, there was evidence of varying activation of numerous circulating cytokines, including KC, G-CSF, IL-6, IL-1β, and IL-12 (Grams and Rabb, 2012). Systemic administration of IL-1β increased TH mRNA expression in brainstem noradrenergic nuclei and increased central NE synthesis, thereby affecting sympathetic efferent activity (Sirivelu et al., 2012). Moreover, renal IRI increased the permeability of the blood-brain barrier (BBB), induced neuronal and microglia damage, and increased levels of pro-inflammatory cytokines including G-CSF, IL-1β in the brain (Liu et al., 2008). The increase in pro-inflammatory cytokines such as TNF-α and IL-1β in PVN activates RAS and ROS in the brain, increasing sympathetic activity (Kang et al., 2008; Shi et al., 2011). Ginsenoside has been shown to have anti-inflammatory and antioxidant effects, and, in AKI model induced by glycerol, the use of ginsenoside attenuated AKI-induced kidney and brain oxidative stress damage and decreased malondialdehyde (MDA) level, a marker of oxidative stress (Mao et al., 2020). Moreover, the use of the central ROS inhibitor tempol attenuated levels of oxidative stress and inflammation as well as renal tissue damage following renal IRI (Cao et al., 2017). The findings further demonstrated the protective effect of blocking central oxidative stress on the kidney. In addition, both systemic and centrally administered AT_1_ receptor blockers, losartan, might have beneficial effects on renal function by reducing serum creatinine and BUN levels (Liu et al., 2008; Sharifi et al., 2019). The main mechanism of its renoprotective effects might be that anti-inflammatory and antioxidant work by blocking the proliferation and activation of leukocytes and chemokines, which were thought to be directly stimulated by Ang II (Vaziri et al., 2007; Crowley and Rudemiller, 2017).

**FIGURE 2:**
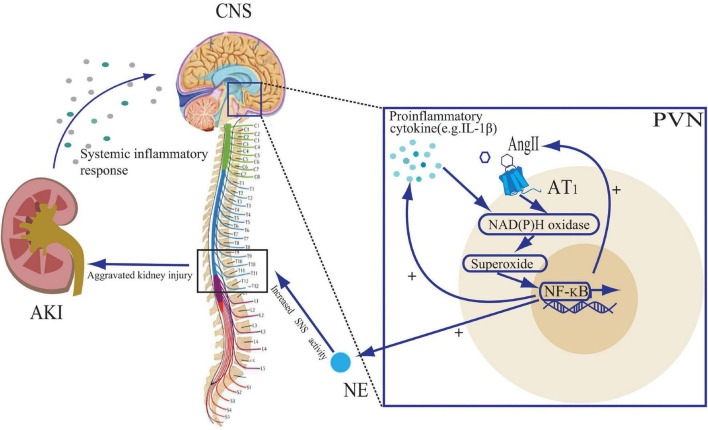
The role of proinflammatory cytokines and angiotensin II (Ang II) in the paraventricular nuclear (PVN) during acute kidney injury (AKI). The systemic inflammatory response to AKI leads to an increase in central nervous system (CNS) pro-inflammatory cytokines. Both Ang II and pro-inflammatory cytokines stimulate NAD(P)H oxidase activity and activate the NF-κB signaling pathway, leading to further cytokine synthesis and Ang II binding to the Ang II type 1 (AT_1_) receptor, creating positive feedback loops. The activation of the NF-κB signaling pathway also leads to an increase in norepinephrine (NE) release, promoting sympathetic nervous system (SNS) activity, further exacerbating kidney injury.

The central action of IL-1β is mediated through the nuclear factor-kappa B (NF-κB) pathway (Nadjar et al., 2003). IL-1β binds to its receptor and undergoes a phosphorylation cascade that translocates NF-κB to the nucleus (Nadjar et al., 2005). In addition, NF-κB activation is facilitated by NAD(P)H oxidase-derived ROS, which are induced by proinflammatory cytokines (Bonizzi et al., 1999; Gloire et al., 2006). An animal study demonstrated that AKI in rats could lead to upregulation of NF-κB and its downstream cyclic oxygenase-2 (COX2)/ prostaglandin E2 (PGE2) expression in brain tissue (Zhang et al., 2014). ROS stimulates COX2 and PGE2 activity via the NF-κB pathway, which in turn stimulates the synthesis of pro-inflammatory cytokines, angiotensinogen and AT1 receptor (Chen and Pan, 2007; Kang et al., 2008). Thus, by acting on the CNS, cytokines and RAS promote each other, creating a positive feedback loop. NF-κB activation promotes inflammatory factor production and NE synthesis in the PVN, while PVN-targeted injection of the NF-κB inhibitor, PDTC, attenuates NF-κB expression and sympathetic nerve activity (Wang et al., 2019). Thus, it could be inferred that central pro-inflammatory factor production due to AKI in turn affects tissue damage therein by increasing central sympathetic activity output, and that inhibition of the CNS inflammation might be a new therapeutic direction (Figure 2).

## The Effect of Acute Kidney Injury on Brain Function

The high mortality rate from AKI is mainly due to extra-renal organ dysfunction (Kelly, 2006). The CNS is more commonly affected by such disease. Many potential uremic toxins, including guanidine-based compounds that regulate nitric oxide synthase (e.g., creatinine, guanidine, and methyl guanidine), have been associated with uremic encephalopathy (Vanholder et al., 2003). Symptoms of encephalopathy are usually more pronounced and progress more rapidly in AKI patients than in patients with CKD and end-stage renal disease (De Deyn et al., 1992). This might be explained by inadequate adaptation time of the uremic toxins accumulated after AKI. A clinical study showed that patients who recovered from AKI had a higher incidence of stroke and mortality than those who did not develop AKI (hazard ratio:1.25) (Wu et al., 2014). Some relevance has been described in animal models of AKI. An experimental study demonstrated that there were reduced dopamine turnover in the striatum, midbrain and hypothalamus of AKI rats, with a reduction in spontaneous motor activity (Adachi et al., 2001). In a mouse model of systemic inflammation, increases in circulating inflammatory cytokines (IL-1β, TNF-α) altered the transcriptional proteins of NF-κB, CCL2, and IL-1β in the hypothalamus and hippocampus (Skelly et al., 2013). In addition, these changes in mice IRI model were shown to result in altered brain function, including a significant increase in fixation neuronal cells in the CA1 region of the hippocampus and an increase in the number of activated microglia and a reduction in its activity (Liu et al., 2008). Interestingly, neuronal sequestration and activated glial cells in the brain were not significantly increased in the corresponding animal model of acute liver injury (Liu et al., 2008), suggesting that this effect is specific to AKI and not just connected with systemic inflammation following organ injuries. A recent study suggested that AKI might lead to hippocampal apoptosis and electrophysiological damage, increased BBB permeability, and memory loss through inflammatory mediators, and that uremia might lead to neuronal necrosis (Firouzjaei et al., 2019).

In addition, it has been shown that renal IRI could lead to oxidative stress in the kidney and brain, and the use of seaweed extract with antioxidant activity could significantly reduce MDA levels in the kidney and hippocampus of rats after IRI to reduce oxidative damage in the kidney and brain (Yu et al., 2016). There were also gender differences in the effects of AKI on brain damage, with the female rats having milder brain damage and MDA levels as compared with the male ones. The differences might be resulted from the protective effect of estrogen by increasing antioxidant enzymes and decreasing inducible NO synthase (iNOS) activity (Azarkish et al., 2021). The level of MDA was significantly decreased in AKI rats treated with losartan intervention. The hyperactivation of AT_1_ receptor by brain Ang II during acute kidney injury has been previously described, and this hyperactivation might precede the release of proinflammatory or inflammatory cytokines in the brain (Saavedra et al., 2011). It appeared that brain inflammation caused by peripheral or central inflammation is closely related to AT_1_ receptor function. In addition, brain MDA levels were significantly reduced in rats with AKI by losartan intervention (Sharifi et al., 2019). Possibly due to the attenuation of neuroinflammation and oxidative stress, losartan-treated rats attenuated AKI-induced memory deficits and impairments (Sharifi et al., 2019). A large US prospective cohort study showed that angiotensin receptor blocker administration could reduce cognitive impairment and Alzheimer’s disease progression (Li et al., 2010). Taken together, these findings suggested that AKI might affect brain function through the production of some toxic metabolites, and through the inflammatory response resulting in BBB destruction and by stimulating inflammation and oxidative stress in the brain. Those processes would lead to modifications in neuronal protein transcription and cell activation.

## Novel Perspectives for Therapeutic Strategies

So far, there were only limited number of measures for treatment of AKI, most of which were supportive therapy and RRT (Vaara et al., 2020). But the mortality rate for AKI patients remained disturbingly high. Targeted treatment developed from the mechanism on how the CNS affects AKI might be a new strategy to improve the effectiveness of AKI treatment and to reduce the occurrence of complications. A retrospective study of 46,253 patients who developed AKI during hospitalization suggested that the use of angiotensin-converting enzyme inhibitor (ACEI) or angiotensin receptor blocker (ARB) might reduce mortality in patients with AKI for two years. This finding could be a potential benefit for AKI patients, but complications of kidney diseases should be carefully monitored in practice (Brar et al., 2018). Similarly, the benefits of ARB have been demonstrated in animal studies. Losartan treatment altered the expression of genes associated with inflammation and oxidative stress in AKI rats, suppressed significant increases in urea and creatinine levels, and reduced tubular structural damage and renal cell apoptosis (Wu et al., 2019). As previously mentioned, activation of the intrarenal RAS in AKI is caused by activation of the central RAS (Campese et al., 2002). Peripherally administered AT_1_ receptor blockers, such as losartan, are able to enter the brain and cause AT_1_ receptor blockade in the CNS, such as the PVN (Wang et al., 2003), thus providing additional therapeutic benefit. In addition, intracerebroventricular administration of losartan to block AT_1_ receptor and tempol to block central oxidative stress in AKI mice both reduced RAS activation, ROS and NE synthesis in the brain, improving brain inflammation, renal injury and dysfunction (Liu et al., 2008). In summary, we could use the blockade of the central RAS and ROS activation, and the control of central inflammation to reduce the damage to the kidney and other organs caused by AKI. Moreover, it was known that AKI could lead to reduced activity of GABAergic neurons in the PVN, and that intravenous GABA treatment or lateral ventricular injection of GABA agonists might reduce renal injury in rats by inhibiting central nervous excitability (Kobuchi et al., 2015). Those findings could also bring new perspective to the treatment of AKI.

## Conclusion

The activation of the CNS due to AKI stimulates the SNS and RAS, exacerbating AKI-induced renal impairment. Therefore, AKI could in turn contribute to the CNS pathology. At present, there were no specific drugs for treatment of AKI. RRT was the only way to minimize the damage, but the mortality for this approach remained high. Recent studies have improved our understanding on the interaction between the CNS and the kidney, with AKI modulating the CNS through a weakening of sympathetic inhibition and a worsening of sympathetic excitation. The end result of this modulation is an increase in central sympathetic outflow, which exacerbates renal injury. Considering the high morbidity and mortality of AKI, there was a requirement to further investigate the mechanisms by which AKI interacts with the nervous system and to develop targeted interventions to reduce the threat of AKI to individuals. By shifting our focus from peripheral to CNS mechanisms, it would help further reduce AKI mortality through use of existing drugs, development of new drugs and application of other treatments with promising perspectives. The safety of drug use, however, must still be taken into account.

## Data Availability Statement

The original contributions presented in the study are included in the article/supplementary material, further inquiries can be directed to the corresponding author/s.

## Author Contributions

QL and YL: conceptualization. YW: methodology and writing—original draft preparation. YW and SL: writing—editing. YL: visualization. QL: supervision. All authors have read and agreed to the published version of the manuscript.

## Conflict of Interest

The authors declare that the research was conducted in the absence of any commercial or financial relationships that could be construed as a potential conflict of interest.

## Publisher’s Note

All claims expressed in this article are solely those of the authors and do not necessarily represent those of their affiliated organizations, or those of the publisher, the editors and the reviewers. Any product that may be evaluated in this article, or claim that may be made by its manufacturer, is not guaranteed or endorsed by the publisher.
